# Antirheumatic therapy is associated with reduced complement activation in rheumatoid arthritis

**DOI:** 10.1371/journal.pone.0264628

**Published:** 2022-02-25

**Authors:** Thao H. P. Nguyen, Ingrid Hokstad, Morten Wang Fagerland, Tom Eirik Mollnes, Ivana Hollan, Mark W. Feinberg, Gunnbjørg Hjeltnes, Gro Ø. Eilertsen, Knut Mikkelsen, Stefan Agewall

**Affiliations:** 1 Lillehammer Hospital for Rheumatic Diseases, Lillehammer, Norway; 2 University of Oslo, Faculty of Medicine, Institute of Clinical Medicine, Oslo, Norway; 3 Department of Laboratory medicine, Innlandet Hospital Trust, Lillehammer, Norway; 4 Oslo Centre for Biostatistics and Epidemiology, Research Support Services, Oslo University Hospital, Oslo, Norway; 5 Department of Immunology, Oslo University Hospital, Oslo, Norway; 6 Research Laboratory, Nordland Hospital, Bodø, and Faculty of Health Sciences, K.G. Jebsen TREC, University of Tromsø, Tromsø, Norway; 7 Centre of Molecular Inflammation Research, Norwegian University of Science and Technology, Trondheim, Norway; 8 Beitostølen Health and Sport Centre, Beitostølen, Norway; 9 Norwegian University of Science and Technology, Gjøvik, Norway; 10 Harvard Medical School, Boston, Massachusetts, United States of America; 11 Division of Cardiology, Brigham and Women´s Hospital, Boston, Massachusetts, United States of America; 12 Department of Internal Medicine, Innlandet Hospital Trust, Lillehammer, Norway; 13 Faculty of Health Sciences, Department of Clinical Medicine, UIT—The Arctic University of Norway, Tromsø, Norway; 14 Department of Rheumatology, University Hospital of North Norway, Tromsø, Norway; 15 Volvat Medical Center, Lillehammer, Norway; 16 Department of Cardiology, Oslo University Hospital, Ullevål, Oslo, Norway; Karolinska Institutet, SWEDEN

## Abstract

**Background:**

The complement system plays an important role in pathophysiology of cardiovascular disease (CVD), and might be involved in accelerated atherogenesis in rheumatoid arthritis (RA). The role of complement activation in response to treatment, and in development of premature CVD in RA, is limited. Therefore, we examined the effects of methotrexate (MTX) and tumor necrosis factor inhibitors (TNFi) on complement activation using soluble terminal complement complex (TCC) levels in RA; and assessed associations between TCC and inflammatory and cardiovascular biomarkers.

**Methods:**

We assessed 64 RA patients starting with MTX monotherapy (n = 34) or TNFi with or without MTX co-medication (TNFi±MTX, n = 30). ELISA was used to measure TCC in EDTA plasma. The patients were examined at baseline, after 6 weeks and 6 months of treatment.

**Results:**

Median TCC was 1.10 CAU/mL, and 57 (89%) patients had TCC above the estimated upper reference limit (<0.70). Compared to baseline, TCC levels were significantly lower at 6-week visit (0.85 CAU/mL, p<0.0001), without significant differences between the two treatment regimens. Notably, sustained reduction in TCC was only achieved after 6 months on TNFi±MTX (0.80 CAU/mL, p = 0.006). Reductions in TCC after treatment were related to decreased C-reactive protein (CRP), erythrocyte sedimentation rate (ESR) and interleukin 6, and increased levels of total, high and low-density lipoprotein cholesterol. Similarly, baseline TCC was significantly related to baseline CRP, ESR and interleukin 6. Patients with endothelial dysfunction had higher baseline TCC than those without (median 1.4 versus 1.0 CAU/mL, p = 0.023).

**Conclusions:**

Patients with active RA had elevated TCC, indicating increased complement activation. TCC decreased with antirheumatic treatment already after 6 weeks. However, only treatment with TNFi±MTX led to sustained reduction in TCC during the 6-month follow-up period. RA patients with endothelial dysfunction had higher baseline TCC compared to those without, possibly reflecting involvement of complement in the atherosclerotic process in RA.

## Introduction

Rheumatoid arthritis (RA) is associated with a wide range of cardiovascular disease (CVD) manifestations. Of those, accelerated atherosclerosis is of particular importance as it is the main cause of premature CVD mediated morbidity and mortality in RA [1–3]. The exact pathogenetic mechanisms behind increased rates of CVD events in RA are still unclear. However, there is evidence that besides traditional CVD risk factors, systemic inflammation and immune dysregulation play a pathogenic role in the development of accelerated atherosclerosis [4–6]. Inflammation alters metabolic processes, and is involved in all stages of the atherosclerotic disease, from induction of endothelial dysfunction, atheroma formation and destabilization, to thrombogenesis [7–9].

Complement is a key component of innate immunity in addition to bridging to adaptive immune responses [10]. It plays a central role in defense system against microbial invaders, clearance of damaged tissue and apoptotic cells, and modulation of inflammatory processes [10,11]. The complement system can be activated via one of three pathways (classical, alternative or lectin), which converge at C3 and C5 activation and share a common terminal pathway. Activation of complement through any of its three activations pathways results in formation of the terminal C5b-9 complement complex, which may remain in plasma as soluble C5b-9 (TCC) or be inserted in the cell membrane as membrane attack complex [10,11]. Excessive activation of the complement system due to chronic inflammatory rheumatic disease, may cause an immune imbalance and increase systemic and local inflammation, and may lead to vascular tissue damage. Inadequate control of complement activation is suggested to play a major role in pathology of inflammatory and autoimmune diseases, including RA, where the cartilage, bone and synovium are targeted [12,13]. High levels of complement activation products have been demonstrated in the synovial fluid and synovial tissues of patients with RA [14–16]. Additionally, RA patients have been reported to have higher concentrations of TCC in the blood than their non-RA counterparts [17–21].

Furthermore, substantial scientific evidence is now available supporting the strong relationship between several complement components and the pathophysiology of numerous CVD outcomes, including coronary artery disease and heart failure [22–26]. TCC promotes activation of platelets and fibrin formation, facilitating thrombogenesis [23]. The complement system is recognized to play an important role in the formation of atherosclerotic lesions, which might result in plaque rupture and thrombus formation with subsequent development of acute coronary syndrome [24–26]. Compared to healthy arteries, complement activation is more pronounced in atherosclerotic arteries, often activated near cholesterol crystals in lesions [27], and in ruptured compared to stable atherosclerotic lesions [28].

However, in spite of the scattered knowledge about the complement system in RA and CVD, little information is available in relation to complement activation in response to treatment and its importance in the development of premature CVD in RA. Therefore, in this study we wanted to investigate the impact of antirheumatic treatment on complement activation in terms of plasma TCC levels in RA. We further assessed associations between TCC levels and established inflammatory and CVD biomarkers, including endothelial function.

## Materials and methods

### Patients

In this study, we included sixty-four patients starting with either methotrexate (MTX) monotherapy (n = 34) or TNF inhibitor (TNFi) with or without MTX co-medication (TNFi ± MTX, n = 30) due to active RA. Patients were recruited as part of the Norwegian prospective observational Psoriatic arthritis, Ankylosing spondylitis, Rheumatoid Arthritis (PSARA) study. Recruitment and study procedures have been described in detail previously [29–31]. Patients fulfilled the American Rheumatism Association 1987 revised criteria for the classification of RA [32], and there was a clinical indication for starting treatment with MTX and/or TNFi (adalimumab, etanercept or infliximab). The treatment modality was based on clinical judgment performed by a rheumatologist not involved in this study, and in accordance with the Norwegian guidelines that adhere to the main international recommendations [33]. All patients starting with MTX were MTX naïve, and all patients starting with TNFi had been previously unsuccessfully treated with MTX.

The study was registered with the following trial registrations: Clinical trials (NCT00902005) and the Norwegian Biobank register (2054). The study was approved by Regional committee for medical and health research ethics in Norway (s-07377b), and all individuals in PSARA gave written informed consent.

### Clinical and laboratory tests

Patients were examined at baseline, after 6 weeks and 6 months of treatment. Data collection included demographic data, medical history, life-style parameters, medication, self-reported questionnaires and physical findings. Venous blood samples were drawn after fasting of eight hours.

Endothelial function was assessed by the Reactive Hyperemia Index (RHI), measured by a fingertip plethysmograph (EndoPAT 2000, Itamar Medical Inc., Caesarea, Israel) as described previously [34,35]. An RH-PAT score of ≤ 1.67 signified endothelial dysfunction, as recommended by the manufacturer [36]. This value of 1.67 was also recommended by the Physiological Diagnosis Criteria for Vascular Failure Committee in the Japan Society for Vascular Failure [37], based on a study of Bonetti et al. [38]. Plethysmography was performed after fasting for at least eight hours, including non-allowance for smoking for at least twelve hours.

### Blood samples

Routine hematologic and biochemical tests including C-reactive protein (CRP), erythrocyte sedimentation rate (ESR), white blood cells (WBC), anti-citrullinated protein antibody (ACPA) and Rheumatoid Factor IgM (RF-IgM) were performed at the local hospital laboratory on the day of collection at all visits. ACPA and RF-IgM were determined by enzyme-linked immunosorbent assays (ELISA), (QUANTA liteTM CCP3 IgG ELISA and QUANTA LiteTM RF IgM ELISA, both INOVA Diagnostics, Inc; San Diego, CA).

Additional blood samples for later analyses (including TCC) were immediately prepared, divided into small aliquots, and stored at -80°C until analyzed.

Complement activation was detected by TCC in EDTA plasma, using our in-house ELISA, as described in detail by Bergseth et al. [39]. Lower detection limit was 0.3 CAU/mL, intra- and inter-assay coefficients of variability were 14% and 15% respectively. The upper reference limit was < 0.7 CAU/mL based on samples form healthy blood donors. The units were designated as Complement Activation Unit (CAU)/mL based on an international standard as described in Bergseth et al. [39].

### Statistical analyses

Continuous data were expressed as median (interquartile range [IQR]) and categorical data by number (percentage) as appropriate. As most continuous variables were not normally distributed, and particularly because the TCC measurements contained several extreme values, median regression was applied for comparisons between and within groups. Median regression is analogous to linear regression but provides inference about medians instead of means. The median regression models used the Hall-Sheather bandwidth method as the nonparametric density estimator under the assumption that the residuals are independent and identically distributed. Spearman’s rank-order coefficients, with 95% confidence intervals based on the Fisher Z transformation, were calculated to evaluate correlations between TCC, RA- and CV related variables at baseline, after 6 weeks and 6 months of antirheumatic treatment. The multiple regression models were adjusted for age and gender and for the baseline characteristics that were statistically significantly related to TCC in simple regression analysis. Chi-squared tests were used for comparisons of categorical data between groups.

A two-tailed probability value of p < 0.05 was considered statistically significant. For statistical calculations we used Stata/SE 16 (StataCorp LLC, College Station, TX).

## Results

### Baseline characteristics of all the patients

Baseline clinical and cardiovascular characteristics of patients are presented in Tables 1 and 2, respectively.

**Table 1:** Baseline clinical characteristics.

	All (n = 64)	MTX (n = 34)	TNFi±MTX (n = 30)	P-value
			
TCC (CAU/mL)	1.1 (0.8–1.5)	1.2 (0.8–2.0)	1.05 (0.8–1.4)	0.71
Age (years)	57.5 (51.5–63)	56.5 (51–63)	59 (55–62)	0.43
Women, n (%)	47 (73.4)	25 (73.5)	22 (73.3)	0.99
**RDD (years)**	**1.5 (0.1–8)**	**0.1 (0.1–1)**	**5 (2–13)**	**0.001**
**Disease activity**				
CRP (mg/L)	8 (3–16)	8 (3–23)	9 (4–14)	0.80
ESR (mm/h)	18.5 (8–29.5)	23 (8–32)	17 (8–27)	0.29
WBC (10⁹/L)	7.3 (5.9–8.6)	7.2 (5.9–8.2)	7.3 (6–8.7)	0.44
RF IgM, n(%)	45 (70.3)	22 (64.7)	23 (76.7)	0.30
ACPA, n(%)	39 (60.9)	17 (50)	22 (73.3)	0.056
IL-6 (ng/L)	18.4 (3.4–52.4)	26.7 (3.6–63.1)	13.8 (3.3–27.9)	0.21
DAS28-ESR	5.1 (4.2–5.5)	4.8 (4.3–5.5)	5.1 (4.1–5.5)	0.37
PtGA	5.2 (3.8–6.7)	5.2 (4.2–6.4)	5.1 (3.3–7.1)	0.90
PGA	3.8 (2.7–4.9)	4 (3.2–4.9)	3.5 (1.9–4.7)	0.57
MHAQ	0.7 (0.3–0.9)	0.5 (0.3–0.9)	0.7 (0.4–1.0)	0.20
Swollen joints	6 (3–9.5)	5.5 (3–10)	6 (3–9)	1.00
**Subcutanous nodules, n(%)**	**12 (18.8)**	**3 (8.8)**	**9 (30)**	**0.03**
**Comedication, n (%)**				
Beta-blockers	5 (7.8)	3 (8.8)	2 (6.7)	0.75
Calcium channel blockers	5 (7.8)	2 (5.9)	3 (10)	0.54
NSAIDs	47 (73.4)	28 (76.5)	21 (70)	0.56
Statins	12 (18.8)	6 (17.7)	6 (20)	0.81
Acetyl salicylic acid	6 (9.4)	4 (11.8)	2 (6.7)	0.49
Glucocorticoids	17 (26.6)	8 (23.5)	9 (30)	0.25

All values are given as median (interquartile range), unless otherwise specified.

Bold values denote statistical significance at the p < 0.05 level.

Abbreviations: MTX: Methotrexate, TNFi: Tumor necrosis factor inhibitors, TCC: Soluble terminal complement complex, RDD: Rheumatic disease duration, CRP: C-reactive protein, ESR: Erythrocyte sedimentation rate, WBC: White blood cells, RF-IgM: Rheumatoid actor immunoglobulin M, ACPA: Anti-citrullinated protein antibody, IL-6: Interleukin 6, DAS28-ESR: Disease Activity Score for 28 joints with ESR, PtGA: Patient’s Global Assessment Score of disease activity, PGA: Physician’s Global Assessment Score of disease activity, MHAQ: Modified Health Assessment Questionnaire, NSAIDs: Non-steroidal anti-inflammatory drugs.

**Table 2:** Baseline cardiovascular characteristics.

	All (n = 64)	MTX (n = 34)	TNFi±MTX (n = 30)	P-value
CVD, n (%)				
Estabished CVD	8 (12.5)	3 (8.8)	5 (16.7)	0.14
Hypertension	17 (26.6)	7 (20.6)	10 (33.3)	0.25
BMI (kg/m^2^)	26.1 (23.6–28.5)	25.6 (23.0–27.2)	27.2 (23.7–32.8)	0.15
Hyperlipidemia	11 (17.2)	7 (20.6)	4 (13.3)	0.44
Current smokers	20 (31.3)	13(38.2)	7 (23.3)	0.20
Diabetes	3 (4.7)	0	3 (10)	0.059
NT-proBNP (ng/L)	77.1 (29.5–127.6)	66.6 (29.6–126.8)	82.8 (23.4–127.6)	0.46
hs-TnT (ng/L)	5 (2.6–7)	5 (2.3–7)	5 (2.9–8)	1.00
**Endothelial function**				
RHI	1.9 (1.6–2.2)	1.8 (1.6–2.0)	1.9 (1.6–2.2)	0.56
ED, n(%)	22 (34.4)	12 (35.3)	10 (33.3)	0.87
**Other characteristics**				
TC (mmol/L)	5.2 (4.6–5.9)	4.9 (4.3–5.7)	5.5 (5.0–6.2)	0.08
LDL-C (mmol/L)	3.2 (2.5–4.0)	3.1 (2.5–3.6)	3.4 (2.7–4.1)	0.35
HDL-C (mmol/L)	1.4 (1.2–1.6)	1.4 (1.1–1.5)	1.4 (1.3–1.7)	1.00
TG (mmol/L)	1.2 (0.95–1.5)	1.2 (0.9–1.5)	1.2 (1.1–1.5)	0.83
Homocystein (μmol/L)	11.5 (10.0–13.5)	12.0 (11.0–14.0)	11.0 (10.0–13.0)	0.20
HbA1C (%)	5.7 (5.5–6.0)	5.7 (5.4–5.9)	5.7 (5.5–6.0)	1.00
FBG (mmol/L)	5.1 (4.9–5.4)	5.1 (5.0–5.4)	5.1 (4.8–5.4)	1.00

All values are given as median (interquartile range), unless otherwise specified.

Established CVD defined as previous presence of any of these conditions: Angina pectoris, stroke, myocardial infarction, carotid stenosis, chronic heart failure, percutaneous coronary angioplasty, aortic aneurysm.

Endothelial dysfunction defined as RHI ≤ 1.67.

Abbreviations: MTX: Methotrexate, TNFi: Tumor necrosis factor inhibitors, CVD: Cardiovascular diseases, BMI: Body mass index, NT-proBNP: N-terminal pro-brain natriuretic peptide, hs-TnT: High-sensitivity troponin T, RHI: Reactive hyperemia index, ED: Endothelial dysfunction, TC: Total cholesterol, LDL-C: Low-density lipoprotein cholesterol, HDL-C: High-density lipoprotein cholesterol, TG: Triglyceride, HbA1C: Glycated hemoglobin, FBG: Fasting blood sugar.

About half of the patients started with MTX monotherapy, while in the other half, TNF±MTX were initiated due to insufficient MTX response. Disease activity was high with a median DAS28-ESR of 5. Patients on TNF±MTX treatment were 4.9 years older than those treated with MTX monotherapy (median disease duration 5.0 versus 0.1 year). More patients in TNF±MTX treatment group had subcutaneous rheumatoid nodules as compared to MTX only treatment group (Table 1).

One third of all the patients smoked, and the number of smokers in the MTX only treatment group did not differ from the TNF±MTX treatment group. No further substantial differences were noted when comparing MTX only treatment group to patients treated with TNFi regimens. (Table 2).

### TCC and RA- and other CVD-related risk factors at baseline

The median baseline TCC level was 1.10 CAU/mL, and 57 (89%) patients had TCC above the upper limit of the estimated reference range with no difference between patients treated with MTX monotherapy and those treated with TNFi regimens (Table 1). No differences in plasma levels of TCC were noted between smokers and non-smokers, neither at baseline (p = 0.19), nor at 6-week (p = 0.21) or 6-month visit (p = 0.47).

**Fig 1:**
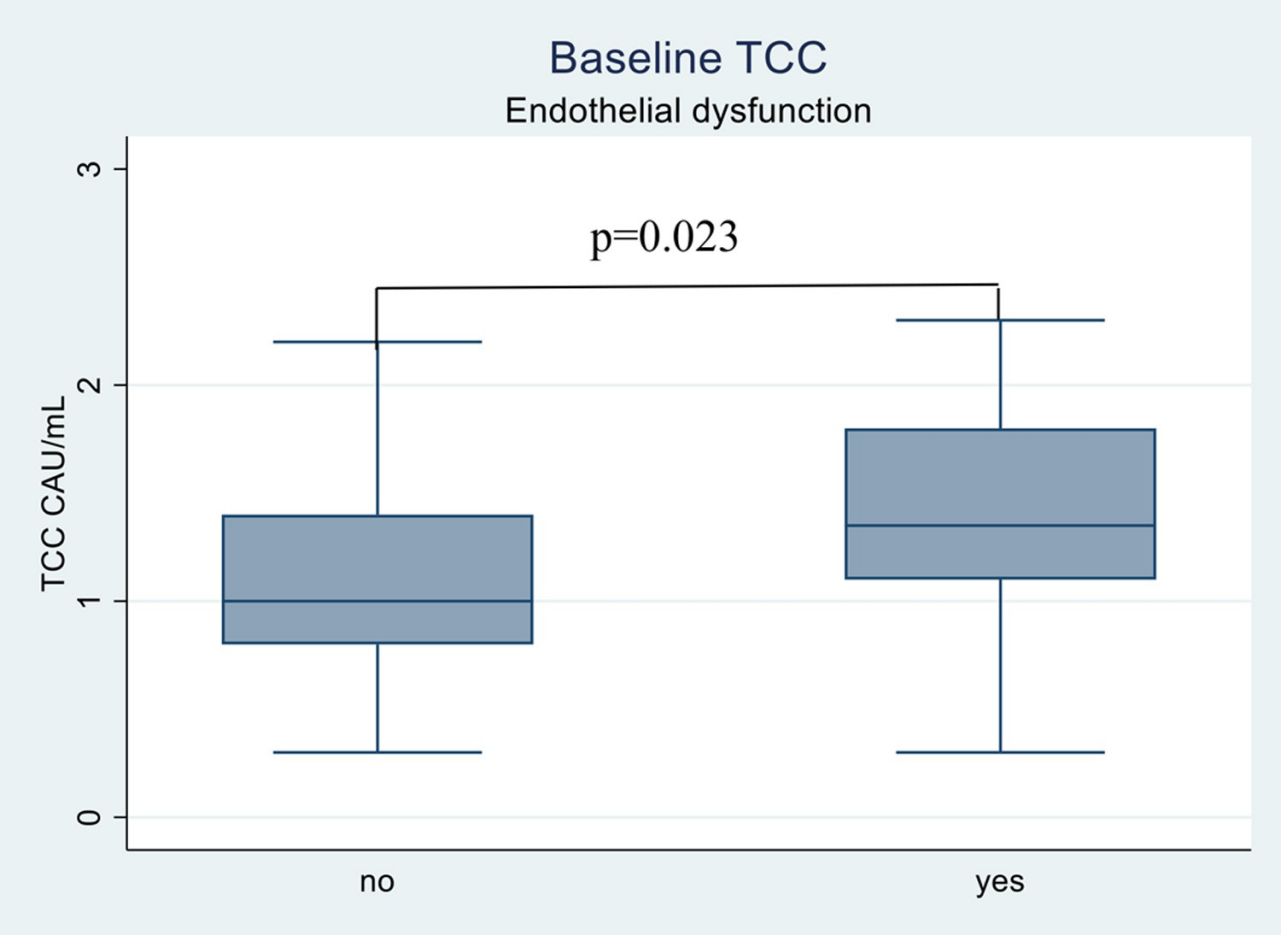
Baseline TCC in patients with and without endothelial dysfunction. Boxplots presenting the distribution of the baseline values of TCC in patients with and without endothelial dysfunction. The lines inside the boxes show the median, bottom and top of the box represent 25 and 75 percentile and whiskers represent minimum and maximum values. Endothelial dysfunction defined as reactive hyperemia index ≤ 1.67. Values are given in median. TCC: Soluble terminal complement complex.

Patients with endothelial dysfunction had significantly higher TCC compared to those without (median 1.4 versus 1.0 CAU/mL, p = 0.023) (Fig 1). However, TCC was not related to RHI at any point of time (p_baseline_ = 0.08, p_6-week_ = 0.82, and p_6-month_ = 0.50), although there was a modestly negative correlation between baseline TCC and baseline RHI in MTX only treatment group (Spearman´s rho = -0.38 [95% CI = -0.63 to -0.04], p = 0.031).

Associations between TCC and selected variables at baseline are presented in Table 3.

**Table 3:** Associations between TCC levels and selected variables at baseline.

	Crude			Multiple median regression		
	Spearman´s rho	95% CI	P-value	Beta	95% CI	P-value
CRP (mg/L)	**0.41**	**0.19 to 0.60**	**0.001**	0.008	-0.003 to 0.02	0.15
ESR (mm/h)	**0.26**	**0.01 to 0.47**	**0.04**	0.005	-0.006 to 0.02	0.36
IL-6 (ng/L)	**0.41**	**0.18 to 0.59**	**0.001**	**0.005**	**0.002 to 0.008**	**0.001**
TNF (ng/L)	-0.15	-0.38 to 0.11	0.26	-0.02	-0.24 to 0.21	0.89
RHI	-0.22	-0.45 to 0.03	0.08	-0.24	-0.70 to 0.23	0.31
PTX3 (ng/mL)	0.18	-0.07 to 0.41	0.16	0.006	-0.07 to 0.08	0.88
NT-proBNP (ng/L)	0.12	-0.13 to 0.35	0.36	0.00004	-0.0003 to 0.0004	0.85
hs-TnT (ng/L)	-0.06	-0.30 to 0.19	0.65	0.003	-0.04 to 0.04	0.99
HbA1C (%)	**0.29**	**0.05 to 0.50**	**0.019**	0.1	-0.18 to 0.39	0.47
HDL-C (mmol/L)	**-0.29**	**-0.50 to -0.04**	**0.022**	-0.31	-0.77 to 0.14	0.18

Bold values denote statistical significance at the p < 0.05 level.

Abbreviations: CRP: C-reactive protein, ESR: Erythrocyte sedimentation rate, IL-6: Interleukin 6, TNF: Tumor necrosis factor, RHI: Reactive hyperemia index, PTX3: Pentraxin 3, NT-proBNP: N-terminal pro-brain natriuretic peptide, hs-TnT: High-sensitivity troponin T, HbA1C: Glycated hemoglobin, HDL-C: High-density lipoprotein cholesterol.

In crude analyses, TCC levels positively correlated with inflammatory markers like CRP, ESR and interleukin 6 (IL-6). However, only association with IL-6 remained significant after adjustment for age and gender (Table 3).

### Changes in TCC levels with antirheumatic treatment

**Fig 2:**
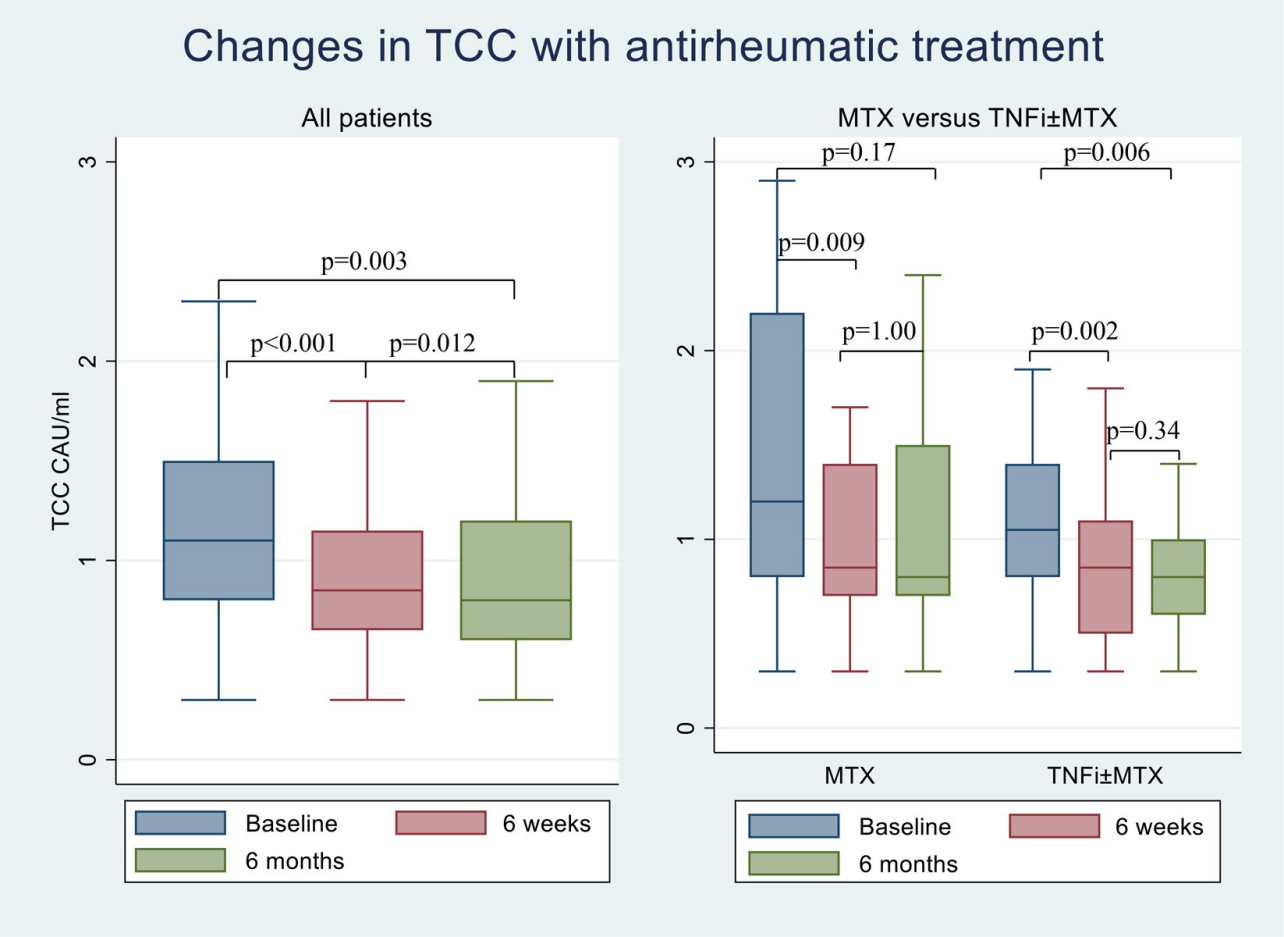
Changes in TCC with MTX monotherapy and TNFi±MTX treatment. Boxplot displaying the difference in TCC at baseline and post-treatment with MTX monotherapy and TNFi±MTX for the whole population. The lines inside the boxes show the median, bottom and top of the box represent 25 and 75 percentile and whiskers represent minimum and maximum values. Values are given in median. MTX: Methotrexate, TNFi: Tumor necrosis factor inhibitors, TCC: Soluble terminal complement complex.

Compared to baseline, TCC levels reduced significantly already after 6 weeks of antirheumatic therapy in both treatment groups. After 6 months on antirheumatic therapy, reduction in TCC persisted only after administration of TNFi±MTX compared to baseline, whereas no significant change was observed in patients treated with MTX only (Figs 2 and S1). However, there were no statistically significant differences in changes in TCC between MTX only group and TNFi±MTX treatment group at any point of time (p_baseline-6weeks_ = 1.00; p_6weeks- i6months_ = 0.34; p_baseline-6months_ = 0.49).

Percentage of patients with TCC above the upper limit of the estimated reference range at 6-week and 6-month visit reduced to 75% and 72%, respectively, as compared to baseline (89%).

In patients with higher TCC levels, antirheumatic treatment led to significant improvement in RHI after 6 weeks, compared to baseline (median 1.84 vs 2.01 CAU/mL, p = 0.029). After 6 months of treatment, a marginal improvement in RHI was observed in MTX only treatment group, as compared to baseline (median 1.80 vs 2.09, p = 0.057).

Changes in TCC and some selected clinical and laboratory variables are shown in Table 4.

**Table 4:** Changes in TCC and some selected clinical and laboratory variables.

	baseline	6w	6m	P(baseline-6w)	P(baseline-6m)
**TCC (CAU/mL)**					
All patients	1.1	0.85	0.8	**<0.001**	**0.003**
MTX	1.2	0.85	0.8	**0.009**	0.17
TNFi±MTX	1.05	0.85	0.8	**0.002**	**0.006**
**CRP (mg/L)**					
All patients	8.0	2.0	2.0	**0.006**	**0.001**
MTX	8.0	3.0	2.0	0.13	0.13
TNFi±MTX	9.0	2.0	2.0	**0.021**	**0.002**
**ESR (mm/h)**					
All patients	18.5	10.5	9.5	**<0.001**	**<0.001**
MTX	23	13	9.5	**0.026**	**0.009**
TNFi±MTX	17	7.5	9.5	**0.014**	**0.001**
**IL6 (ng/L)**					
All patients	18.4	3.9	2.7	**0.011**	0.058
MTX	26.7	5.7	2.0	0.25	**0.023**
TNFi±MTX	13.8	2.1	3.6	**0.048**	0.13
**DAS28-ESSR**					
All patients	5.0	3.4	2.5	**<0.001**	**<0.001**
MTX	4.9	3.8	2.6	**<0.001**	**<0.001**
TNFi±MTX	5.0	3.1	2.4	**<0.001**	**<0.001**
**MHAQ**					
All patients	0.65	0.3	0.2	**0.023**	**<0.001**
MTX	0.48	0.25	0.13	0.12	**0.003**
TNFi±MTX	0.7	0.4	0.2	**0.002**	**<0.001**
**PGA**					
All patients	3.9	2.3	1.4	**<0.001**	**<0.001**
MTX	4.0	2.7	1.9	**0.002**	**<0.001**
TNFi±MTX	3.8	2.0	1.3	**0.003**	**<0.001**
**PtGA**					
All patients	5.2	2.9	1.5	**<0.001**	**<0.001**
MTX	5.2	3.0	1.4	**<0.001**	**<0.001**
TNFi±MTX	5.1	2.4	1.6	**0.007**	**<0.001**

Bold values denote statistical significance at the p < 0.05 level.

Abbreviations: MTX: Methotrexate, TNFi: Tumor necrosis factor inhibitors, TCC: Soluble terminal complement complex, CRP: C-reactive protein, ESR: Erythrocyte sedimentation rate, IL-6: Interleukin 6, DAS28-ESR: Disease Activity Score for 28 joints with ESR, MHAQ: Modified Health Assessment Questionnaire, PGA: Physician’s Global Assessment Score of disease activity, PtGA: Patient’s Global Assessment Score of disease activity.

### Associations of changes in TCC with markers related to arthritis activity and CVD

Associations of changes in TCC with selected clinical and laboratory variables after 6 weeks and 6 months of antirheumatic treatment are shown in Tables 5 and 6, respectively.

**Table 5:** Associations between changes in TCC and changes in selected markers of arthritis activity after 6 weeks of treatment.

	Crude			Multiple median regression		
	Spearman´s rho	95% CI	P-value	Beta	95% CI	P-value
**ΔCRP**						
All	**0.41**	**0.18 to 0.60**	**0.0008**	**0.01**	**0.002 to 0.02**	**0.018**
MTX	0.20	-0.15 to 0.50	0.26	0.01	-0.02 to 0.04	0.40
TNF±MTX	**0.67**	**0.40 to 0.83**	**0.0001**	0.008	-0.003 to 0.02	0.15
**ΔESR**						
All	**0.36**	**0.13 to 0.56**	**0.003**	**0.01**	**0.003 to 0.02**	**0.004**
MTX	0.27	-0.08 to 0.55	0.13	0.007	-0.09 to 0.02	0.41
TNF±MTX	**0.48**	**0.14 to 0.71**	**0.008**	0.01	0.001 to 0.02	0.075
**ΔIL-6**	** **	** **	** **			
All	**0.31**	**0.06–0.52**	**0.015**	0.002	-0.0003 to 0.003	0.10
MTX	0.12	-0.25 to 0.46	0.49	-0	-0.007 to 0.004	0.54
TNF±MTX	**0.61**	**0.32 to 0.80**	**0.0004**	0.007	-0.0007 to 0.02	0.073
**ΔDAS28-ESR**		** **	** **			
All	0.10	-0.15 to 0.34	0.43	0.06	-0.03 to 0.15	0.20
MTX	-0.16	-0.47 to 0.19	0.38	-0.02	-0.16 to 0.11	0.72
TNF±MTX	**0.49**	**0.16 to 0.72**	**0.006**	**0.21**	**0.08 to 0.33**	**0.003**
**ΔMHAQ**						
All	0.22	-0.03 to 0.45	0.079	0.21	-0.07 to 0.50	0.14
MTX	0.16	-0.19 to 0.48	0.37	0.01	-0.81 to 0.84	0.97
TNF±MTX	0.34	-0.02 to 0.62	0.065	**0.32**	**0.04 to 0.59**	**0.026**
**PGA**						
All	0.09	-0.16 to 0.33	0.48	0.03	-0.03 to 0.08	0.37
MTX	-0.16	-0.47 to 0.19	0.37	-0.03	-0.24 to 0.18	0.80
TNF±MTX	**0.41**	**0.05 to 0.67**	**0.026**	0.07	-0.004 to 0.14	0.061

Bold values denote statistical significance at the p < 0.05 level.

Δ indicates change from baseline to 6 weeks.

Abbreviations: MTX: Methotrexate, TNFi: Tumor necrosis factor inhibitors, TCC: Soluble terminal complement complex, rho: Spearman’s rho, CRP: C-reactive protein, ESR: Erythrocyte sedimentation rate, IL-6: Interleukin 6, DAS28-ESR: Disease Activity Score for 28 joints with ESR, MHAQ: Modified Health Assessment Questionnaire, PGA: Physician’s Global Assessment Score of disease activity.

**Table 6:** Associations between changes in TCC and changes in selected variables after 6 months of treatment.

	Crude			Multiple median regression		
	Spearman´s rho	95% CI	P-value	Beta	95% CI	P-value
**ΔCRP**						
All	**0.42**	**0.20 to 0.60**	**0.0006**	**0.008**	**0.003 to 0.01**	**0.004**
MTX	0.32	-0.02 to 0.60	0.063	0.008	-0.04 to 0.06	0.74
TNF±MTX	**0.62**	**0.33 to 0.80**	**0.0003**	0.008	-0.0004 to 0.02	0.062
**ΔESR**						
All	**0.35**	**0.11 to 0.54**	**0.005**	0.005	-0.001 to 0.01	0.11
MTX	0.23	-0.12 to 0.53	0.19	0.004	-0.04 to 0.05	0.87
TNF±MTX	**0.58**	**0.28 to 0.78**	**0.0007**	0.01	-0.002 to 0.02	0.097
**ΔIL-6**	** **	** **	** **			
All	0.03	-0.22 to 0.27	0.84	**0.003**	**0.001 to 0.004**	**0.001**
MTX	-0.04	-0.37 to 0.30	0.82	0.003	-0.001 to 0.008	0.15
TNF±MTX	0.26	-0.12 to 0.57	0.17	0.003	-0.003 to 0.001	0.29
**ΔDAS28-ESR**		** **	** **			
All	**0.27**	**0.02 to 0.48**	**0.033**	**0.10**	**0.009 to 0.18**	**0.031**
MTX	0.21	-0.14 to 0.51	0.24	0.02	-0.38 to 0.43	0.91
TNF±MTX	0.36	-0.01 to 0.63	0.054	0.12	-0.03 to 0.27	0.12
**ΔMHAQ**						
All	**0.33**	**0.09 to 0.53**	**0.008**	0.36	-0.10 to 0.81	0.12
MTX	0.28	-0.06 to 0.57	0.11	0.53	-2.06 to 3.12	0.68
TNF±MTX	**0.46**	**0.12 to 0.70**	**0.011**	0.21	-0.50 to 0.92	0.55
**ΔPtGA**	** **	** **	** **			
All	**0.33**	**0.01 to 0.53**	**0.007**	0.04	-0.007 to 0.09	0.097
MTX	0.32	-0.02 to 0.59	0.064	0.02	-0.22 to 0.26	0.88
TNF±MTX	0.36	-0.002 to 0.64	0.052	**0.07**	**0.02 to 0.13**	**0.015**
**ΔTC**						
All	**-0.34**	**-0.54 to 0.11**	**0.005**	-0.10	-0.26 to 0.06	0.23
MTX	-0.31	-0.59 to 0.03	0.076	-0.10	-1.68 to 1.49	0.90
TNFi	**-0.45**	**-0.70 to -0.11**	**0.011**	-0.15	-0.43 to 0.13	0.28
**ΔHDL-C**						
All	-0.17	-0.39 to 0.08	0.19	-0.31	-0.80 to 0.18	0.21
MTX	0.09	-0.26 to 0.41	0.63	0.12	-2.25 to 2.49	0.92
TNF±MTX	**-0.61**	**-0.79 to -0.31**	**0.0004**	**-1.00**	**-1.60 to -0.40**	**0.002**
**ΔLDL-C**						
All	**-0.36**	**-0.56 to -0.13**	**0.003**	-0.12	-0.34 to 0.10	0.29
MTX	-0.32	-0.59 to 0.02	0.067	-0.26	-2.04 to 1.52	0.77
TNF±MTX	**-0.40**	**-0.67 to -0.05**	**0.028**	-0.12	-0.47 to 0.23	0.49
**ΔTG**						
All	**-0.36**	**-0.56 to -0.12**	**0.003**	-0.27	-0.66 to 0.12	0.17
MTX	**-0.60**	**-0.78 to -0.33**	**0.0002**	-1.38	-5.78 to 3.02	0.52
TNF±MTX	0.12	-0.25 to 0.46	0.52	-0.05	-0.65 to 0.55	0.87

Bold values denote statistical significance at the p < 0.05 level.

Δ indicates change from baseline to 6 months.

Abbreviations: MTX: Methotrexate, TNFi: Tumor necrosis factor inhibitors, TCC: Soluble terminal complement complex, rho: Spearman’s rho, CRP: C-reactive protein, ESR: Erythrocyte sedimentation rate, IL-6: Interleukin 6, DAS28-ESR: Disease Activity Score for 28 joints with ESR, MHAQ: Modified Health Assessment Questionnaire, PtGA: Patient’s Global Assessment Score of disease activity, TC: Total cholesterol, HDL-C: High-density lipoprotein cholesterol, LDL-C: Low-density lipoprotein cholesterol, TG: Triglyceride.

Reductions in TCC from baseline to 6 weeks and from baseline to 6 months were related to decreased CRP, ESR and IL-6.

Reduction in TCCC after 6 months on antirheumatic treatment was correlated with decrease of some clinical markers of RA activity (e.g., DAS28-ESR, PtGA and MHAQ), and with increased levels of all types of cholesterol, and triglycerides (Table 6).

At 6-month visit, it was observed a marginal elevation in total cholesterol level (median difference = 0.20 [95% CI = -0.006 to 0.41], p = 0.056), and a statistically significant increase in HDL-C (median difference = 0.10 [95% CI = 0.02 to 0.18], p = 0.012) in the whole population. Changes in LDL-C and triglyceride did not reach statistical significance level (median difference = 0.10 [95% CI = -0.05 to 0.25], p = 0.20 and median difference = -0.02 [95% CI = -0.13 to 0.09], p = 0.72, respectively)

## Discussion

In this study, complement activation as detected by plasma TCC was increased in the majority of patients with active RA, and the complement activation reduced rapidly and sustainably after initiation of antirheumatic treatment. Notably, during the 6-month follow-up period, sustained reduction in TCC was achieved only after administration of TNFi±MTX, whereas treatment with MT alone reduced TCC after 6 weeks, but not after 6 months. TCC positively correlated to inflammatory markers at baseline, and reductions in TCC with treatment were related to decreased inflammatory markers and increased total cholesterol, high-density lipoprotein (HDL-C) and low-density lipoprotein cholesterol (LDL-C) values. Moreover, baseline TCC levels were significant lower in RA patients with normal endothelial function compared to those with disturbed endothelial function.

The presence of increased complement activation in autoimmune arthritis, such as RA and spondyloarthropathies, has been reported before [14–16,40], and is confirmed by the results of the present study. This might point to the contribution of complement activation in the pathogenesis of RA.

Concordant with previous studies, our data showed a clear association between complement activation and markers of RA disease activity, and that initiation of TNFi brought on sustained reduction in complement activation in form of TCC, as well as these inflammatory markers [40,41–43]. However, the exact pathogenetic mechanisms regarding complement activation in response to TNFi therapy are still incompletely understood. Several explanations may partly account for this.

First, up to 90% of plasma complement components are produced by hepatocytes [44], and hepatic synthesis is upregulated in response to various cytokines, e.g., TNF [45–47], which is a pivotal mediator and driver of inflammation in RA [48]. This may explain the finding of increased soluble complement products in our patients, reverted by inhibition of TNF.

Second, former studies have demonstrated that the classical pathway of complement is in part activated by CRP [42,49,50], a routinely assessed marker of systemic inflammation in RA [51]. Therefore, reduction in CRP induced by antirheumatic therapy, could be associated with decreased complement activation.

Third, antirheumatic therapy exert their effect partly by inhibition of IL-6-production, leading to downregulation of CRP synthesis by hepatocytes and subsequently reduced activation of the classical pathway [52–54].

It is worth mentioning that MTX monotherapy also led to rapid reduction in TCC already within 6 weeks, but at 6 months, the change in TCC did not reach statistical significance. There are few studies suggesting any direct effect of MTX on complement system. The anti-inflammatory effects of MX are broad, and the mechanisms are not fully understood. MTX, via its metabolite adenosine, exerts inhibitory effect on production of TNF and promote secretion of IL-6, and thus might attenuate the complement cascade [55]. Moreover, MTX exposure has been shown to be associated with higher expression of complement genes, and increased complement activation products C3 and C5 in liver in patients with inflammatory arthritis [56].

Thus, the observed data in our study are consistent with the conclusion that reduction of complement system could be one of the mechanisms by which TNFi exert their effectiveness on RA. Our findings suggest that TCC could be an attractive biomarker for measurement of disease activity and also effect of TNFi in RA.

There is strong scientific evidence for the involvement of inflammation in endothelial dysfunction, the subclinical precursor to overt CVD [57,58]. Mechanistically, TCC activation and the membrane attack complex can initiate NLRP3 inflammasome activation triggering induction of IL-1ß, IL-6, and IL-18 secretion in endothelial cells, vascular smooth muscle cells, or leukocytes in the vessel wall [59,60]. Our result ties well with previous studies wherein patients with endothelial dysfunction had significant higher circulating levels of TCC in the blood [61,62], and that endothelial function improved with antirheumatic treatment (data not completely shown) [63,64].

Taken together, our findings support the notion that complement activation is involved in chronic inflammation and in endothelial dysfunction, and suggest that better control of complement activation might also control atherogenesis and ameliorate CVD risk.

However, in the present study, we did not find correlation between change in TCC and change in endothelial function after treatment. This might reflect the complexity and not fully elucidated pathophysiological mechanisms of endothelial dysfunction in our patients. The lack of association between TCC and endothelial function after 6 months on antirheumatic treatment could be due to our method of endothelial function assessment. Several tools have been validated for assessing vascular endothelial function, and the most commonly performed and best validated method is brachial artery ultrasound scanning (BAUS), evaluating flow-mediated dilatation (FMD) [65]. This technique, recommended by the international Brachial Artery Reactivity Task Force, is based on the percentage change of the brachial artery diameter during reactive hyperemia. Nevertheless, accurate assessment of FMD is technically challenging to perform, and this method is strongly operator-dependent, and therefore prone to operator bias and high variability [66]. The reactive hyperemia-peripheral arterial tonometry (RH-PAT) test, based on the same principle of reactive hyperemia as BAUS, is relatively simple, easy to perform in a standardized manner, less operator-dependent than that of FMD, and with comprehensive automatic analysis. Another benefit of RH-PAT is that this technique evaluates vascular dilatation in both arms–using contralateral arm as its internal control to correct for systemic changes during testing [34,35]. However, the main disadvantage of this technique is that it could be difficult to obtain stable measurements because RH-PAT recordings have shown to be sensitive to mental stress, anxiety, smoking and hyperglycemia [67–69]. To minimize the impact of confounding factors on the RH-PAT results, our patients were examined in tightly controlled environments, including rest period of 15 minutes, fasting for at least eight hours and refraining from tobacco for at least twelve hours before examination.

Moreover, no associations were observed between TCC and circulating markers for CVD, including coronary artery disease and heart failure, such as high-sensitivity troponin T, N-terminal pro-brain natriuretic peptide and pentraxin 3.

High levels of systemic inflammation in RA are associated with suppression of total cholesterol, LDL-C and HDL-C levels, partly due to impaired cholesterol efflux capacity of HDL-C [70,71]. Our data showed that reductions in TCC after 6 months of antirheumatic treatment were correlated with increased levels of all types of cholesterol and triglycerides. The results are in accordance with a former study demonstrating an inverse correlation of TCC and HDL-C [70], and that disease modifying antirheumatic drugs are associated with increased LDL cholesterol and triglyceride levels, possibly due to inhibition of inflammation [72,73]. Nonetheless, the increased cholesterol levels after treatment do not indicate increased atherogenicity as cholesterol quality and cholesterol transport, including HDL-C efflux capacity, may improve with treatment [73,74]. Increase of HDL-C level after antirheumatic treatment may also contribute to reduction in TCC as HDL-C carries many proteins that inhibit complement activation [75], and HDL-C can impair the cytolytic activity of TCC [27].

Cigarette smoking is supposed to enhance complement cleavage products, as it induces a pro-inflammatory environment specifically by generating complement factors C3a and C3b [76,77]. Nevertheless, in our study, there was no association between TCC and tobacco smoke, neither pre- nor post antirheumatic treatment. The results are in line with former study showing no change in TCC expression in sera exposed to tobacco smoke extract, despite signs of upstream complement activation [78]. Our patients were instructed not to smoke in at least twelve hours prior to examination, and it is unclear whether temporary smoking cessation affects outcomes.

The findings of this study have to be seen in lights of some limitations including the relatively small sample size and the observational design. The former may increase the risk of Type-II errors, and might lead to underestimation of real differences and associations. Moreover, it is not ethically appropriate to withhold treatment with beneficial effect from clinically eligible patients. The statistical approach utilized in our research paper also suffers from the limitation that multiple testing of longitudinal data amplifies the probability of a false-positive finding.

An advantage of our study is the comprehensive characterization of patient population and the design that makes it possible to compare the effect of the two antirheumatic treatment regimens on TCC levels in RA.

In conclusion, RA patients had elevated TCC, which decreased rapidly and sustainably with TNFi±MTX treatment during the 6-month period. Exaggerated complement activation due to chronic inflammation in RA, seems to be associated with endothelial dysfunction–the initial step in the pathogenesis of atherosclerosis. Our findings support further investigations to determine if the cardioprotective effects of antirheumatic drugs might be associated to ameliorating effects of TNFi on complement activation, and whether the complement system might be a target for therapy in RA.

## Supporting information

S1 Fig Scatterplots. Changes in TCC with MTX monotherapy and TNFi±MTX treatment.
(TIF)

S1 File Stata file. Stata file containing all data underlying the findings described in the present study.
(DTA)
