# Supplementary figures and images for: Antirheumatic therapy is associated with reduced complement activation in rheumatoid arthritis
Source: PLoS One. 2022 Feb 25;17(2):e0264628. doi: 10.1371/journal.pone.0264628 (PMC8880951; doi:10.1371/journal.pone.0264628)

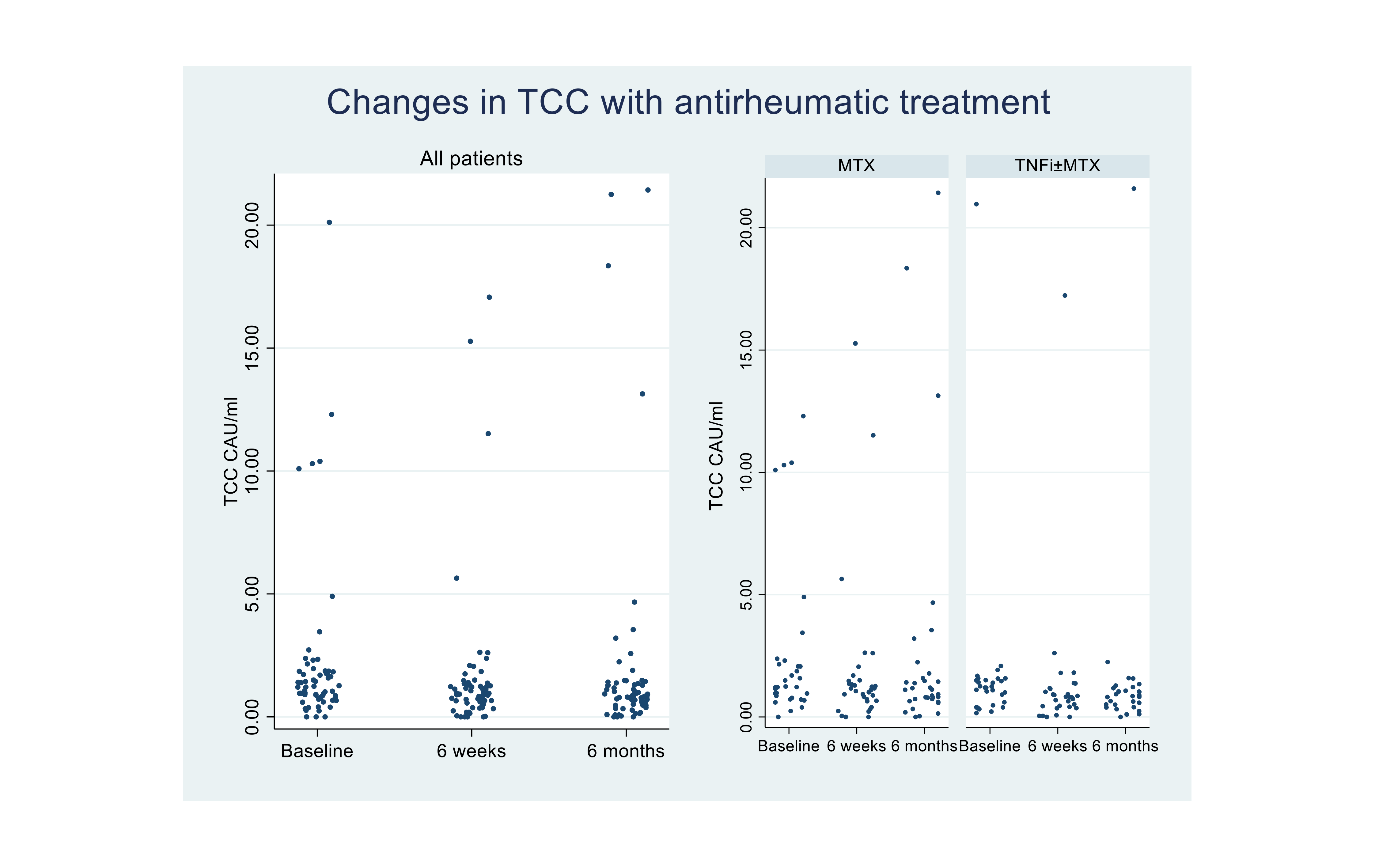

Supplement: S1 Fig — Changes in TCC with MTX monotherapy and TNFi±MTX treatment. (TIF) [file pone.0264628.s001.tif]
